# Robotic-assisted seminal vesiculectomy for treatment of refractory hematospermia

**DOI:** 10.1016/j.eucr.2023.102541

**Published:** 2023-08-21

**Authors:** Matthew Satariano, Olivia Green, Neel Parekh

**Affiliations:** a Northeast Ohio Medical University, 4209 St. Rt. 44, PO Box 95, Rootstown, OH, 44272, United States; b Cleveland Clinic Akron General, 1 Akron General Avenue, Akron, OH, 44307, United States

**Keywords:** Seminal vesiculectomy, Hematospermia, Robotic surgery, Seminal vesicles, 5-alpha reductase inhibitor

## Abstract

Hematospermia is a common, but anxiety-provoking genitourinary condition. In instances without spontaneous resolution, pharmacologic intervention with a 5-alpha reductase inhibitor has been shown to be successful. In cases of refractory hematospermia, robotic-assisted laparoscopic seminal vesiculectomy may provide a definitive treatment option. A robotic-assisted bilateral seminal vesiculectomy was performed on a 42-year-old male with refractory painless hematospermia after failing conservative management. Three months post-operatively, the patient reported resolution of hematospermia after six ejaculations with no impact on erectile function. The robotic-assisted approach is safe and feasible with good functional outcomes and reduced morbidity.

## Highlights

• Hematospermia can be caused by a variety of genitourinary conditions.
• 5-alpha reductase inhibitors may be used to treat refractory hematospermia.
• 5-alpha reductase inhibitors may not be tolerated due to sexual side effects.
• Robotic seminal vesiculectomy successfully treats refractory hematospermia.

## 1 Introduction

Hematospermia is a common symptom associated with genitourinary conditions such as seminal vesicle cysts or stones, urogenital infections, testicular cancer (seminoma), congenital anomalies, benign prostatic hyperplasia (BPH), or systemic disease. Typically, hematospermia is self-limiting, only requiring observation and reassurance. In cases without spontaneous resolution, pharmacologic intervention with finasteride (5-alpha reductase inhibitor) has been shown to decrease the amount, depth, and frequency of bleeding.**^1^** But, in some cases of refractory hematospermia, seminal vesiculectomy may be necessary for definitive treatment. A laparoscopic approach was first introduced by Kavoussi et al., in 1993**^2^** and has since been successfully reported. In 2002, a partial seminal vesiculectomy was successfully performed to treat a patient with a seminal vesicle cyst and subsequent hematospermia.**^3^** In 2019, a seminal vesiculectomy was performed to treat a patient with Zinner syndrome, a congenital abnormality of the Wolffian (mesonephric) duct which results in a triad of symptoms (unilateral renal agenesis, ejaculatory duct obstruction, and ipsilateral seminal vesicle cyst).**^4^** Here we report a case of refractory hematospermia in a 42-year-old male in which robotic seminal vesiculectomy was successfully performed for symptom resolution.

## 2 Case presentation

**Fig. 1:**
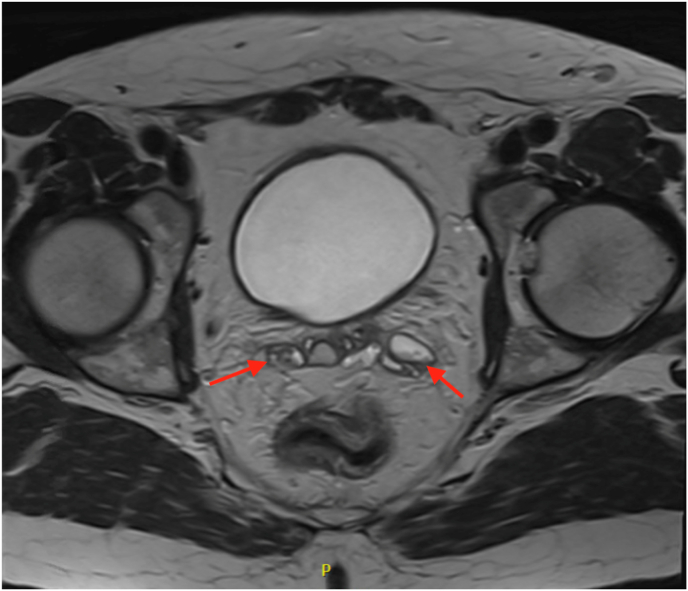
Hemorrhagic cyst (8 × 13 × 8 mm) at the base of the right seminal vesicle. Cyst containing debris and fluid (19 × 10 × 8 mm) within left seminal vesicle. Indicated by the arrows.

**Fig. 2:**
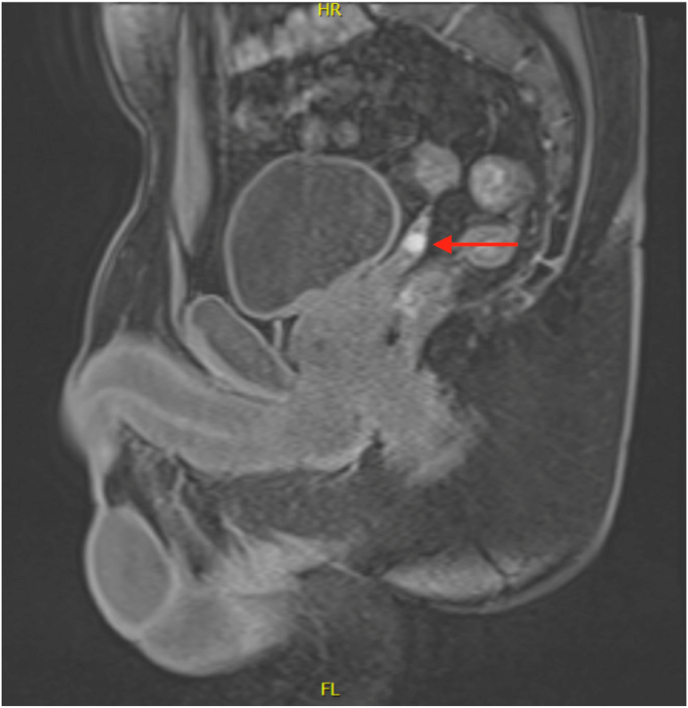
Sagittal view of right seminal vesicle hemorrhagic cyst (8 × 13 × 8 mm). Indicated by arrow.

**Fig. 3:**
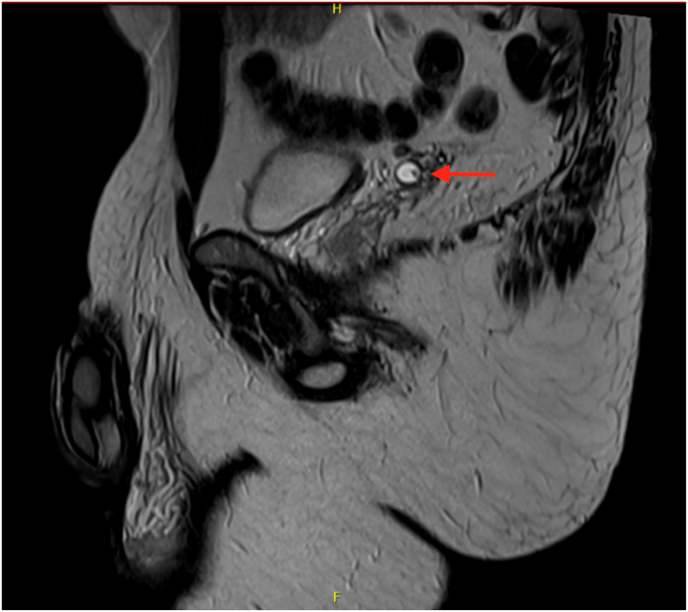
Sagittal view of left seminal vesicle cyst (19 × 10 × 8 mm). Indicated by arrow.

A 42-year-old Caucasian male presented with a six-week history of painless hematospermia. His past urological history involved uneventful vasectomy with excision of an incidental right spermatic cord mass in 2018. Final pathology demonstrated a benign fibrous pseudotumor. His current medications included doxepin and lisdexamfetamine. His social history included drinking four beers per week, no smoking history, and no pertinent family history. The patient's physical examination, including digital rectal exam, was normal. He had been prescribed doxycycline with no improvement of symptoms before being referred to urology. A sexual transmitted disease (STD) panel, including Chlamydia, Mycoplasma, HIV, Syphilis, and Hepatitis, was negative. Prostate-specific antigen (PSA) was 0.7 ng/mL. Urinalysis showed no evidence of infection or microhematuria. At his one-month follow-up, the patient reported several episodes of gross hematuria and continued hematospermia. Scrotal ultrasound (SUS) revealed a shadowing extratesticular mass superior to the right testicle, that appeared separate from the epididymal head, and a left-sided varicocele. The patient subsequently underwent CT Urography which showed no abnormalities. Urine culture and cytology were also negative. Office cystoscopy revealed a cystic lesion arising within the verumontanum. Transrectal ultrasound (TRUS) revealed a cystic and dilated appearance of the right seminal vesicle. The persistent hematospermia was likely contributing to reduced libido and psychogenic erectile dysfunction. However, a hormone panel was obtained, and testosterone, luteinizing hormone, prolactin, estradiol 17B, and thyroid stimulating hormone were within normal range. Given the abnormal TRUS, Magnetic resonance imaging (MRI) of the pelvis with IV contrast was obtained. In the base of the right seminal vesicle, an 8 × 13 × 8 mm T1 hypointense density was noted, consistent with hemorrhagic cyst (Fig. 1, Fig. 2). In the left seminal vesicle, there was a 1.9 × 1.0 × 0.8 cm, likely debris, ovoid density (Fig. 1, Fig. 3). Initiation of pharmacologic therapy with 5-alpha reductase inhibitor was deferred due to the patient's concerns about the sexual side effects and potential lack of efficacy in this setting.

Given the intractable nature of the hematospermia and psychological toll being placed on the patient and his partner, the couple was interested in pursuing surgical intervention. The couple was not interested in future fertility potential.

Six months after the patient's initial visit, a robotic-assisted laparoscopic bilateral seminal vesiculectomy was performed. The posterior peritoneum was incised and the right vas deferens identified and mobilized distally towards the prostate. The right seminal vesicle was identified and mobilized, taking care to avoid electrocautery laterally near the neurovascular bundle. The seminal vesicle and vas were then fully mobilized posteriorly to the level of the prostatic capsule. The procedure was repeated on the left side. Following complete posterior mobilization, a plane was developed anteriorly between the prostate and the seminal vesicles and vasa deferentia. This was similarly carried to the level of the prostatic adenoma and capsule. The specimens were then excised and sent to pathology. Pathology revealed a benign epithelial cyst on the right seminal vesicle and a dilated left seminal vesicle with inspissated secretions. Three months post-operatively, the patient reported resolution of hematospermia after six ejaculations and no issues with erections.

## 3 Discussion

Hematospermia is a common symptom associated with genitourinary conditions such as seminal vesicle cysts or stones, urogenital infections, testicular cancer (seminoma), congenital anomalies, BPH, or systemic disease. In cases without spontaneous resolution, pharmacologic intervention with finasteride has been shown to decrease the amount, depth, and frequency of bleeding in patients with hematospermia.**^1^** In some cases of refractory hematospermia, seminal vesiculectomy may be necessary to treat the condition such as in patients with seminal vesical cysts, testicular seminomas, or Zinner syndrome. The increased use of TRUS and CT have helped in making this diagnosis.**^5^** In addition, primary malignancy of the seminal vesicles, which are very rare, could be noted on imaging and would be an indication for surgical intervention.

In our case, several conservative, noninvasive approaches were first taken to treat the condition. Empiric oral antibiotic administration yielded no improvement of symptoms, and a STD panel, PSA, and urine studies were negative. MRI revealed a right seminal vesicle hemorrhagic cyst (Fig. 1, Fig. 2) and left seminal vesicle cysts containing debris with mild dilatation of left seminal vesicle ducts (Fig. 1, Fig. 3). Seminal vesicle cysts may be indicative of obstruction and visualizing blood products within the seminal vesicle on MRI helps localize the seminal vesicle as the culprit. Around 90% will be self-limiting and resolve over time conservatively. So, while cysts are not an absolute indication or criteria for surgical intervention, cysts, calculi and tumors are associated with hematospermia and may lead to need for intervention. Additionally, MRIs often show changes in the seminal vesicle with hematospermia, and bleeding can take time to clear out. To address this, it is important to obtain a proper history and physical such as noting a prior pelvic malignancy. If the patient is less than 40 years old with no risk factors, no further investigation is needed. If the patient is over 40 years old, there are risk factors, or it is refractory, it is recommended to obtain PSA, TRUS, or MRI of the pelvis. It can be done as soon as 3 months if the patient is significantly bothered. Serial ejaculation more than 15 times is recommended over those 3 months to try and clear blood products.

Initiation of finasteride was recommended for our patient; but, he declined this option due to concerns of sexual side effects. A robotic-assisted bilateral seminal vesiculectomy was thus performed based on previous literature detailing its success in treating refractory hematospermia.^2^^,^^3^ But, it is important to note the sexual side effects of seminal vesiculectomy. One study looked at mice with either bilateral seminal vesicle occlusion (SVO), bilateral seminal vesicle resection (SVR), or sham operation (SO) and analyzed the number of sessions of intromission among the groups.^6^ The highest percentage of intromission occurred in the SVO group with subsequent engorgement of the seminal vesicles, suggesting an intrinsic role of the seminal vesicles in sexual activity. While our patient did not have sexual side effects thus far, this is one area to investigate in the future as there is limited research available.

In the past, conventional surgery has been proposed for access to the seminal vesicles. However, the various approaches have been associated with postoperative pain and morbidity.**^5^** The laparoscopic approach has been associated with decreased postoperative pain, less negative side effects, and successful treatment of symptoms.**^4^** Thus, the robotic-assisted approach was taken for our patient and had successful outcomes with less morbidities.

## 4 Conclusion

Thorough workup of recurrent hematospermia is important to identify or exclude underlying pathology. Hematospermia is a common symptom associated with various genitourinary conditions or systemic disease. While it is typically self-limiting, in cases of hematospermia refractory to conservative management, robotic-assisted seminal vesiculectomy is an effective option.

## Funding

This research did not receive any specific grant from funding agencies in the public, commercial, or not-for-profit sectors.

## Consent

The proper consent was obtained for this research.

## Section headings

Male Lower Urinary Tract Symptoms.

## Declaration of competing interest

There are no conflicts of interest.

## References

[1] Badawy A.A., Abdelhafez A.A., Abuzeid A.M. (2012 Apr). Finasteride for treatment of refractory hemospermia: prospective placebo-controlled study. Int Urol Nephrol.

[2] Kavoussi L.R., Schuessler W.W., Vancaillie T.G., Clayman R.V. (1993 Aug). Laparoscopic approach to the seminal vesicles. J Urol.

[3] Manousakas T., Kyriakou G., Serafetinides E., Giannopoulou M., Kyroudi A., Giannopoulos A. (2002 Apr). Partial vesiculectomy in an infertile man with seminal vesicle cyst, ipsilateral renal agenesis, and cryptorchidism. Urology.

[4] Corongiu E., Grande P., Olivieri V., Pagliarella G., Forte F. (2019 Mar 29). Minimally invasive management of a symptomatic case of Zinner's syndrome: laparoscopic seminal vesiculectomy and ipsilateral nephroureterectomy. Arch Ital Urol Androl.

[5] Ploumidis A., Sooriakumaran P., Philippou P., Wiklund N.P. (2012). Robotic-assisted laparoscopic vesiculectomy for lower urinary tract obstruction by a large seminal vesicle cyst. Int J Surg Case Rep.

[6] Birkhäuser F.D., Schumacher C., Seiler R. (2012). Occlusion of seminal vesicles increases sexual activity in a mouse model. Eur Urol.

